# Methylene blue-induced Hemolysis in a patient with lidocaine-induced Methemoglobinemia: a case report

**DOI:** 10.1093/omcr/omaf264

**Published:** 2025-12-26

**Authors:** Murad Mehmood, Fatema Jaber A Almarri

**Affiliations:** Department of Emergency Medicine, Hamad General Hospital, Al Rayyan Road, Hamad Medical City (Zone 37), Ad Dawhah (Doha Municipality), State of Qatar, P.O. Box 3050, Doha 16060, Qatar; Department of Emergency Medicine, Hamad General Hospital, Al Rayyan Road, Hamad Medical City (Zone 37), Ad Dawhah (Doha Municipality), State of Qatar, P.O. Box 3050, Doha 16060, Qatar

**Keywords:** Methemoglobinemia/chemically induced, Hemolysis/chemically induced, methylene blue/adverse effects, lidocaine/toxicity, ascorbic acid/therapeutic use

## Abstract

Background: Methemoglobinemia is an uncommon but potentially life-threatening disorder where hemoglobin iron is oxidized to the ferric state, impairing oxygen transport. Topical anesthetics such as lidocaine can induce methemoglobinemia. Methylene blue (MB) is the first-line antidote, but high doses can oxidize hemoglobin and may precipitate hemolysis even in individuals with normal glucose-6-phosphate dehydrogenase (G6PD) activity. Case Presentation: A 31-year-old woman with recurrent genital herpes self-applied excessive 5% lidocaine cream (≈3 tubes/day) for two weeks. She presented with progressive fatigue, cyanosis and hypoxemia. Arterial blood gas showed methemoglobin 13.6%. She received intravenous MB at another hospital and subsequently developed severe anemia (hemoglobin 6.6 g/dL) with laboratory evidence of intravascular hemolysis. G6PD activity was normal. Supportive management included high-flow oxygen, oral ascorbic acid, intravenous antivirals and antibiotics, and transfusion of two units of packed red blood cells. Methemoglobin levels and hemoglobin gradually normalized, and she was discharged in stable condition. Conclusion: This case illustrates the paradoxical risk of MB-induced hemolysis in a G6PD-sufficient patient. Clinicians should consider alternative treatments such as ascorbic acid when managing methemoglobinemia and educate the public on safe use of topical anesthetics.

## Introduction

Methemoglobinemia occurs when the ferrous iron of hemoglobin is oxidized to ferric iron (methemoglobin) that cannot bind oxygen, and severe cases may be life-threatening [1, 2]. Increased methemoglobin shifts the oxygen–hemoglobin dissociation curve to the left, causing functional anemia and tissue hypoxia [2]. Congenital forms arise from cytochrome b_5_ reductase deficiency, while acquired forms result from exposure to oxidizing agents including nitrates, dapsone, and local anesthetics such as lidocaine, benzocaine, prilocaine and tetracaine [2–4]. Topical anesthetics can induce methemoglobinemia after mucosal or cutaneous application; lidocaine is less commonly implicated than benzocaine but remains a recognized cause [3, 4]. Clinical clues include cyanosis unresponsive to oxygen therapy, chocolate-colored blood, and a saturation gap between arterial blood gas and pulse oximetry. When methemoglobin levels exceed 20% or symptoms are significant, antidotal therapy is indicated [2].

Methylene blue reduces methemoglobin via NADPH-dependent pathways, converting it to leucomethylene blue which then reduces ferric iron back to the ferrous state. At high doses, however, MB can oxidize hemoglobin, paradoxically exacerbating methemoglobinemia and precipitating hemolysis [5]. Hemolytic episodes are well recognised in G6PD-deficient individuals, but rare cases have been reported in G6PD-sufficient patients [6, 7]. We report a case of MB-associated hemolysis following treatment for lidocaine-induced methemoglobinemia in a patient with normal G6PD activity.

## Case report

A previously healthy 31-year-old woman presented to the Emergency Department with progressive fatigue, central cyanosis and persistent hypoxemia. She had a history of recurrent genital herpes simplex virus infection and had self-applied approximately three tubes of over-the-counter 5% lidocaine cream per day to the genital area for two weeks to control pain. She initially attended a private hospital where oxygen saturation on room air was 80%. Arterial blood gas (ABG) analysis demonstrated methemoglobin 13.6%; she received intravenous MB (dose unknown) and was transferred to our tertiary centre for ongoing management.

On arrival she was conscious and oriented but tachypnoeic (respiratory rate 26 breaths per minute) with oxygen saturation 84% on room air, improving only to 86% with a non-rebreather mask. She was clinically cyanotic and had generalized pallor but no bleeding or organomegaly. Laboratory investigations revealed hemoglobin 6.6 g/dL, markedly elevated reticulocyte count (17.7%), elevated lactate dehydrogenase and indirect bilirubin, undetectable haptoglobin and normal Coombs test, indicating intravascular hemolysis. G6PD activity was within the normal range. Table 1 summarizes the complete blood count and metabolic panel. ABG on room air showed pH 7.41, PaO₂ 123 mmHg, PaCO₂ 39 mmHg and methemoglobin 13.6% (Table 2). Imaging, including chest radiograph, abdominal ultrasound and computed tomography of the abdomen and pelvis, revealed no bleeding or collections.

**Table 1:** Laboratory data at presentation. The complete blood count showed severe anemia (hemoglobin 6.6 g/dL), elevated mean corpuscular volume and marked reticulocytosis, while the metabolic panel demonstrated mild elevation of bilirubin and normal renal function.

Test	Patient Value	Units
Complete Blood Count (CBC)		
Hemoglobin (Hb)	6.6	g/dL
Hematocrit (Hct)	19.9	%
MCV	103.6	fL
MCH	34.4	pg
MCHC	33.2	g/dL
RDW-CV	31.6	%
White Blood Cells (WBC)	26.3	×10^3^/μL
Platelets	361	×10^3^/μL
Neutrophils	22.1	%
Lymphocytes	1.8	%
Retic #	352.6	×10^3^/μL
Retic %	17.7	%
Comprehensive Metabolic Panel (CMP)		
Sodium (Na^+^)	135	mmol/L
Potassium (K^+^)	4	mmol/L
Chloride (Cl^−^)	104	mmol/L
Bicarbonate (HCO₃^−^)	22	mmol/L
Blood Urea Nitrogen (BUN)	1.3	mmol/L
Creatinine	55	μmol/L
Glucose	5.7	mmol/L
Calcium (Ca^2+^)	2.06	mmol/L
Bilirubin Total	30	μmol/L
Albumin	28	g/L
Total protein	54	g/L
ALT (Alanine Aminotransferase)	16	U/L
ALP (Alkaline Phosphatase)	55	U/L
CRP	15.7	mg/L
G6PD	Normal	

**Table 2:** Arterial blood gas on room air showing normal pH and PaO₂ but elevated methemoglobin fraction (13.6%) and reduced O₂-hemoglobin saturation.

Test	Patient Value	Units
pH	7.41	
PO₂	123	mmHg
PCO₂	39	mmHg
Methemoglobin	13.6	%
O₂-Hemoglobin	77.6	%
CO-Hemoglobin	5	%
SO₂	95.3	%
Bicarbonate (HCO₃^−^)	24.7	mmol/L
Lactate	0.6	mmol/L

A working diagnosis of lidocaine-induced methemoglobinemia complicated by MB-associated hemolysis was made. She was admitted for close monitoring. Treatment consisted of high-flow oxygen, oral ascorbic acid 2 g four times daily as an alternative reducer, and intravenous acyclovir for herpetic infection. Two units of packed red blood cells were transfused for symptomatic anemia. Empiric broad-spectrum antibiotics were started and later de-escalated based on culture results. Over several days her symptoms resolved and laboratory indices improved. Methemoglobin levels normalized with conservative management. She was discharged after completing a 7-day course of antivirals and antibiotics and remained well at two-week follow-up.

## Discussion

Lidocaine is an amide local anesthetic widely used for topical analgesia. In overdose, it can undergo oxidative metabolism to anilide metabolites that oxidize hemoglobin to methemoglobin, especially when applied to mucosal surfaces or ulcerated skin. Although prilocaine is more potent in this regard, lidocaine-induced methemoglobinemia is well documented [3, 4]. Our patient applied a large topical dose over an extended period, likely facilitating systemic absorption and oxidative stress. Clinical features of methemoglobinemia include cyanosis refractory to oxygen therapy, dyspnea, headache and, at high levels, confusion and arrhythmias. Pulse oximetry values around 85% regardless of supplemental oxygen and chocolate-colored blood suggest the diagnosis [2].

Methylene blue is recommended first-line therapy when methemoglobin levels exceed 20% or patients are symptomatic [2]. MB acts as an artificial electron acceptor, becoming reduced to leucomethylene blue which then reduces methemoglobin via NADPH methemoglobin reductase. However, at high doses MB can act as an oxidant and paradoxically induce methemoglobinemia [5]. Furthermore, MB is known to precipitate hemolysis in individuals with G6PD deficiency because their red blood cells cannot generate sufficient NADPH to reduce MB [5]. Literature suggests that MB may cause mild decreases in hemoglobin even in G6PD-normal individuals; pooled analyses and case reports have demonstrated small reductions in hemoglobin and rare episodes of hemolysis [6, 7]. Similar adverse effects have been described following exposure to benzocaine and other topical anesthetics [8–10]. The presented case, along with isolated reports, indicates that MB-induced hemolysis can occur despite normal G6PD activity, possibly owing to individual variability in oxidative stress responses or unrecognized enzymopathies. In our patient, the temporal association between MB administration and sudden hemolysis, absence of bleeding, and normalization after discontinuation support MB as the culprit.

Management of methemoglobinemia in patients at risk of hemolysis should be individualized. Ascorbic acid (vitamin C) serves as an alternative treatment for methemoglobinemia, particularly in patients where methylene blue is contraindicated (e.g. G6PD deficiency or oxidative hemolysis). It acts as a non-enzymatic reducing agent, directly donating electrons to convert ferric (Fe^3+^) methemoglobin back to functional ferrous (Fe^2+^) hemoglobin, thereby restoring oxygen-carrying capacity. Though slower in onset than methylene blue, repeated high doses can achieve clinically meaningful reductions in methemoglobin [2]. Other therapeutic options include exchange transfusion and hyperbaric oxygen for refractory cases. Prevention is equally important: clinicians must educate patients about the risks of excessive topical anesthetic use, particularly over large surface areas, and ensure that MB dosing follows recommended guidelines. In our patient, inadvertent overuse of lidocaine precipitated methemoglobinemia, and subsequent MB administration exacerbated hemolysis. Public education and clinician awareness can prevent such events.
